# Supplementary material for: Neonatal brain injury influences structural connectivity and childhood functional outcomes
Source: PLoS One. 2022 Jan 5;17(1):e0262310. doi: 10.1371/journal.pone.0262310 (PMC8730412; doi:10.1371/journal.pone.0262310)
Supplement: S1 Table — (DOCX) [file pone.0262310.s001.docx]

| **S1 Table. MRI injury patterns in hypoxic-ischemic encephalopathy.** | | | | |  |
| --- | --- | --- | --- | --- | --- |
|  | **Basal Ganglia Injury Score** | | | | |
|  | **Score 0**  *Normal or isolated focal cortical infarct* | **Score 1**  *Abnormal signal in the thalamus* | **Score 2**  *Abnormal signal in the thalamus and lentiform nucleus* | **Score 3**  *Abnormal signal in the thalamus, lentiform nucleus, and perirolandic cortex* | **Score 4**  *More extensive involvement* |
| **Watershed Injury Score** – n (%) |  |  |  |  |  |
| **Score 0**  *Normal* | 40 (66.7%) | 1 (1.7%) | 2 (3.3%) | - | - |
| **Score 1**  *Single focal infarct* | 8 (13.3%) | - | - | - | - |
| **Score 2**  *Abnormal signal in anterior or posterior*  *watershed white matter* | 2 (3.3%) | 1 (1.7%) | - | - | - |
| **Score 3**  *Abnormal signal in anterior or posterior*  *watershed cortex and white matter* | 4 (6.7%) | - | - | - | - |
| **Score 4**  *Abnormal signal in anterior and posterior*  *watershed zones* | 2 (3.3%) | - | - | - | - |
| **Score 5**  *More extensive cortical involvement* | - | - | - | - | - |
