# Supplementary material for: Neonatal brain injury influences structural connectivity and childhood functional outcomes
Source: PLoS One. 2022 Jan 5;17(1):e0262310. doi: 10.1371/journal.pone.0262310 (PMC8730412; doi:10.1371/journal.pone.0262310)
Supplement: S2 Table — (DOCX) [file pone.0262310.s002.docx]

**S2 Table. MRI injury patterns in congenital heart disease.**

|  | **SV**  n = 20 | **TGA**  n = 15 | **All**  n = 35 |
| --- | --- | --- | --- |
| **Brain Injury Score – n (%)** |  |  |  |
| 0: Normal | 13 (65%) | 9 (60%) | 22 (62.9%) |
| 1: Mild white matter injury | 1 (5%) | 2 (13.3%) | 3 (8.6%) |
| 2: Stroke | 1 (5%) | 3 (20%) | 4 (11.4%) |
| 3: Moderate-severe white matter injury | 5 (25%) | 1 (6. 7%) | 6 (17.1%) |
