# Supplementary material for: Neonatal brain injury influences structural connectivity and childhood functional outcomes
Source: PLoS One. 2022 Jan 5;17(1):e0262310. doi: 10.1371/journal.pone.0262310 (PMC8730412; doi:10.1371/journal.pone.0262310)
Supplement: S3 Table — A. Neurodevelopmental outcomes in those with and without injury on MRI. B. Neurodevelopmental outcomes in those with SV and TGA. (DOCX) [file pone.0262310.s003.docx]

**S3A Table. Neurodevelopmental Outcomes in those with and without injury on MRI.**

|  | **HIE**  **n = 60** | | | **CHD**  **n = 35** | | | **All** |
| --- | --- | --- | --- | --- | --- | --- | --- |
|  | **Normal MRI**  n = 40 (66.7%) | **Abnormal MRI**  n = 20 (33.3%) |  | **Normal MRI**  n = 22 (62.9%)  SV 13, TGA 9 | **Abnormal MRI**  n = 13 (37.1%)  SV 7, TGA 6 |  | **Normal MRI vs. Abnormal MRI** |
| **Bayley-III Score**  **– Median (IQR)** |  |  | **P** |  |  | **P** | **Median, P** |
| 12-18 mo. Cognitive | 107.5 (95-112.5)  *n = 29* | 100 (95-112.5)  *n = 13* | 0.41 | 97.5 (85-115)  *n = 10* | 100 (95-113.75)  *n = 4* | 0.67 | 105 vs. 100, 0.61 |
| 12-18 mo. Language | 103 (97-112)  *n = 29* | 110.5 (103-115)  *n = 13* | 0.07 | 90 (79-94)  *n = 10* | 87.5 (86-89)  *n = 4* | 0.60 | 97 vs. 106, 0.22 |
| 12-18 mo. Motor | 98.5 (91-107)  *n = 29* | 97 (86.5-107)  *n = 13* | 0.82 | 94.75 (91-100)  *n = 10* | 88 (76-106)  *n = 4* | 0.43 | 97 vs. 92.5, 0.66 |
| 30 mo. Cognitive | 100 (95-113.75)  *n = 20* | 100 (95-112.5)  *n = 12* | 0.53 | 90 (68-95)  *n = 7* | 95 (95-95)  *n = 1* | 0.75 | 95 vs. 100, 0.16 |
| 30 mo. Language | 98.5 (84.5-115)  *n = 20* | 98.5 (89-112)  *n = 11* | 0.68 | 91 (63.5-91)  *n = 7* | 83 (83-83)  *n = 1* | 1.00 | 91 vs. 97.75, 0.49 |
| 30 mo. Motor | 98.5 (97-110)  *n = 20* | 100.5 (91-109.3)  *n = 12* | 0.79 | 91 (80.5-97)  *n = 7* | 91 (91-91)  *n = 1* | 1.00 | 97 vs. 94, 0.80 |

**S3B Table. Neurodevelopmental Outcomes in those with SV and TGA.**

|  | **Single Ventricle**  n = 20 | **Transposition**  n = 15 |  |
| --- | --- | --- | --- |
| **Bayley III Score – Median (IQR)** |  |  | **p** |
| 12-18 mo. Cognitive | 92.5 (85-105)  *n = 6* | 100 (95-126.25)  *n = 8* | 0.21 |
| 12-18 mo. Language | 90 (86-94)  *n = 6* | 87.5 (82.5-91.5)  *n = 8* | 0.47 |
| 12-18 mo. Motor | 89.5 (64-100)  *n = 6* | 94.75 (91-100.75)  *n = 8* | 0.29 |
| 30 mo. Cognitive | 75 (55-95)  *n = 2* | 90 (80-95)  *n = 6* | 0.79 |
| 30 mo. Language | 77.25 (63.5-91)  *n = 2* | 87 (83-91)  *n = 6* | 0.86 |
| 30 mo. Motor | 69.25 (47.5-91)  *n = 2* | 92.5 (85-97)  *n = 6* | 0.36 |
