# Supplementary material for: Neonatal brain injury influences structural connectivity and childhood functional outcomes
Source: PLoS One. 2022 Jan 5;17(1):e0262310. doi: 10.1371/journal.pone.0262310 (PMC8730412; doi:10.1371/journal.pone.0262310)
Supplement: S4 Table — A. Graphics metrics in those with and without injury on MRI. B. Graphics metrics in single ventricle and transposition of the great arteries. (DOCX) [file pone.0262310.s004.docx]

**S4A Table. Graphics metrics in those with and without injury on MRI.**

|  | **HIE**  n = 60 | |  | **CHD**  n = 35 | |  | **All** |
| --- | --- | --- | --- | --- | --- | --- | --- |
|  | **Normal**  n = 40 | **Injury**  n = 20 |  | **Normal**  n = 22 | **Injury**  n = 13 |  | **Normal vs. injury** |
| **Graph metric – Median (IQR)** |  |  | **p** |  |  | **p** | **Medians,**  **p-value** |
| Global efficiency | 0.22  (0.20 - 0.23) | 0.20  (0.19 - 0.22) | 0.08 | 0.20  (0.19 - 0.21) | 0.21  (0.19 - 0.22) | 0.75 | 0.21 vs. 0.20,  p = 0.15 |
| Modularity | 0.19  (0.15 - 0.20) | 0.16  (0.15 - 0.23) | 0.93 | 0.18  (0.16 - 0.20) | 0.16  (0.15 - 0.19) | 0.11 | 0.18 vs. 0.16,  p = 0.27 |
| Transitivity | 0.34  (0.32 - 0.37) | 0.33  (0.30 - 0.35) | 0.18 | 0.32  (0.31 - 0.35) | 0.34  (0.32 - 0.36) | 0.23 | 0.33 vs. 0.33,  p = 0.75 |
| Small-worldness | 1.09  (1.05 -1.15) | 1.08  (1.03 - 1.21) | 0.98 | 1.09  (1.06 - 1.15) | 1.06  (1.03 - 1.11) | 0.06 | 1.09 vs. 1.07,  p = 0.20 |

**S4B Table. Graphics metrics in single ventricle and transposition of the great arteries.**

|  | **Single Ventricle**  n = 20 | **Transposition**  n = 15 |  |
| --- | --- | --- | --- |
| **Graph metric – Median (IQR)** |  |  | **p** |
| Efficiency (FA) | 0.21 (0.19 - 0.21) | 0.20 (0.18 - 0.22) | 0.81 |
| Modularity (FA) | 0.17 (0.15 - 0.20) | 0.18 (0.15 - 0.20) | 0.83 |
| Transitivity (FA) | 0.33 (0.32 - 0.35) | 0.32 (0.31 -0.35) | 0.71 |
| Small Worldness (FA) | 1.07 (1.05 -1.15) | 1.08 (1.04 -1.11) | 0.90 |
