# Supplementary material for: Neonatal brain injury influences structural connectivity and childhood functional outcomes
Source: PLoS One. 2022 Jan 5;17(1):e0262310. doi: 10.1371/journal.pone.0262310 (PMC8730412; doi:10.1371/journal.pone.0262310)
Supplement: Attachment — Submitted filename: Response to Reviewers.docx [file pone.0262310.s005.docx]

Professor of Pediatrics & Neurology

1550 4^th^ Street, RM RH-548D

San Francisco, CA 94143-2711

Phone: 415-502-4798	Fax: 415-514-0235

Email: patrick.mcquillen@ucsf.edu

Monday, October 25, 2021

PLOS
1265 Battery Street, Suite 200
San Francisco, CA 94111
United States

Dear Professor Duerden and PLOS ONE staff,

We are gratified that both reviewers found our paper to be ‘very interesting’ and ‘well written’. Below we respond to all queries raised by the reviewers.

The major issue common to both reviewers relates to the need for a stronger justification of comparing two clinical cohorts without a cohort of healthy controls. It is unfortunate that the two cohorts compared in this paper do not have contemporary matched normal controls. Mitigating this issue is the fact that all neurodevelopmental outcome assessments used in the paper are based upon population standards, thereby providing a context for each cohort in comparison to the general population. An additional strength of the study is that the imaging methods were identical for each cohort. To justify the comparison, we note that the two conditions (congenital heart disease-CHD and hypoxic ischemic encephalopathy-HIE) are among the highest risk for neonatal brain injury, but that injury occurs with very distinct timing and mechanism in each. In CHD, disrupted fetal circulation leads to impaired brain oxygen and substrate delivery throughout the duration of the 3^rd^ trimester with additional risk for injury before and after surgery. In contrast, HIE results from sub-acute or acute pathology in the days/weeks or hours before birth. Comparing the two populations provides a unique natural ‘experiment’ akin to studies in animal models varying injury timing, mechanism, and severity to determine cell populations and systems with selective vulnerability and mechanisms influencing functional outcomes including repair and plasticity. Our results identify distinct differences between the two cohorts that will be of broad general interest to pediatricians, psychologists, and neuroscientists. The CHD cohort has worse functional outcomes overall, especially language, and a unique pattern of hypoconnectivity in specific language associated circuits. At the same time, a metric of overall network topology (global efficiency) is strongly associated with motor outcomes in both cohorts!

We have extensively re-written the introduction to emphasize these points and provide a stronger justification for comparing two clinical cohorts. In addition, we have also addressed the journal requirements – we have ensured that our manuscript meets the PLOS One style requirements, we have added supplemental tables to make transparent data that previously was not shown, and we have ensured that the ‘Funding Information’ and ‘Financial Disclosure’ sections match. All other reviewer queries have been addressed with modifications described below.

Reviewer #1

*P.9 line 121, the two newborns who did not receive TH should be excluded from the analyses. I understand that the results may not have been different, but they are just inducing noise considering that they are not meeting the neat inclusion criteria. Particularly the one with mild HIE and considering that none had neurodevelopmental follow-up.*

These two subjects have been removed and all analyses redone without their data.

*Was CP an exclusion criteria? I was surprised to see no case of suspected CP reported in 42 HIE.*

Cerebral palsy was not an exclusion criterion. The birth asphyxia cohort was prospectively enrolled and all eligible subjects with suitable neonatal imaging data and neurodevelopmental follow-up were included. We believe the beneficial effects of therapeutic hypothermia account for the reduced rate of cerebral palsy. An alternative explanation is that subjects with severe cerebral palsy did not return for subsequent neurodevelopmental evaluations, however in our cohort we did not see a difference in follow-up rates between those with and without brain injury on imaging to support that hypothesis.

*p.17, line 280: Did the CHD patients performed within the normal range for the cognitive and motor domains as well?*

The individual data points for both the CHD and HIE cohorts as well as general population means and standard deviations are provided in figure 3. CHD patients largely performed within one standard deviation of the normal range, and we have added this to the results section to better clarify that finding.

*p.19 line 315: I recommend being careful when using the term predictor when presenting univariate associations between neurodevelopmental outcome and clinical factors, this study is not powered for this.*

We have replaced the term predictor in the univariate association analyses.

*p.21 second paragraph: What are the results of the multivariate analysis and outcomes at 12-18months?*

We did not perform a multivariate analysis of outcomes at 12-18 months because the structural connectivity metrics were not found to be associated with outcomes in the univariate analysis at this time point. We have added to the results section to better clarify that finding and decision.

*p.25 line 432: “transitivity predicted motor outcomes at 2.5 years of life but not earlier at 12-18 months.” Do you mean at 30 months (not 2.5 years)?*

We have replaced 2.5 years with 30 months throughout the manuscript for consistency.

Reviewer #2

*The study in my opinion would benefit greatly from a control group, instead of comparing two types of inducers of neonatal brain injuries directly to each other. I see that the groups are sporadic compared to reference data but in general the two groups are being compared to each other.

If it is a possibility, I would recommend doing the comparison with a healthy control group. However, there is some merit in the question of which is worse; CHD or HIE?*

These are valid points and we have endeavored to address them as delineated above.

Thank you for your consideration,

Sincerely,

Patrick McQuillen (corresponding author) & Alice Ramirez (first author)
